# Beyond Symptom Control: Surgical Treatment of Benign Prostatic Hyperplasia and Reduced Risks of Depression, Anxiety, and Dementia

**DOI:** 10.1192/j.eurpsy.2026.11578

**Published:** 2026-07-31

**Authors:** S.-K. Kao, W.-Y. Cheng, Y.-T. Yu, W.-H. Tseng

**Affiliations:** 1 Psychiatry, Taipei Veterans General Hospital, Taipei; 2 Urology, Chi Mei Medical Center, Tainan; 3 Community Medicine, Landseed International Hospital, Taoyuan; 4 Biomedical Science, National Sun Yat-Sen University, Kaohsiung, Taiwan

## Abstract

**Introduction:**

Benign prostatic hyperplasia (BPH) is a common urological condition in aging men, often causing lower urinary tract symptoms (LUTS). LUTS are linked not only to impaired quality of life but also to psychiatric disorders, sleep disturbances, and cognitive decline. While surgical treatment is widely used to relieve bladder outlet obstruction, its long-term impact on psychiatric and cognitive outcomes remains unclear.

**Objectives:**

To evaluate whether surgical treatment for BPH is associated with reduced risks of psychiatric disorders, sleep disturbances, cognitive decline, and persistent LUTS.

**Methods:**

A retrospective cohort study was conducted using the TriNetX Global Collaborative Network (155 healthcare organizations). Male patients aged ≥18 years with BPH (2015–2024) were included. Patients with baseline psychiatric disorders were excluded. The study population was divided into surgical (n = 20,328) and non-surgical (n = 1,659,904) cohorts. After 1:1 propensity score matching, 20,323 patients remained in each group. Outcomes within three years included depression, anxiety, insomnia, dementia, delirium, and LUTS. Hazard ratios (HR) and p-values were calculated. Subgroup analyses were performed by age and BPH medication use.

**Results:**

Surgical patients had lower risks of persistent LUTS (HR 0.917, p < 0.001), depression (HR 0.616, p < 0.001), anxiety (HR 0.551, p < 0.001), insomnia (HR 0.645, p < 0.001), dementia (HR 0.705, p = 0.013), and delirium (HR 0.694, p = 0.007). Benefits were more pronounced in men aged 18–70 for LUTS, depression, anxiety, and insomnia, whereas men >70 mainly benefited for depression, anxiety, and dementia. Protective effects were stronger in patients not taking medications.

**Table 1.** Risk of Psychiatric Disorders and LUTS in BPH Patients Undergoing Surgery versus Non-Surgical Controls (BPH = Benign Prostatic Hyperplasia; LUTS = Lower Urinary Tract Symptoms; HR = Hazard Ratio; CI = Confidence Interval; * p < 0.05; *** p < 0.001) Table 1. Long description.

Outcome	Surgery (N=20,323)	non-Surgery (N= 20,323)	Hazard ratio (95% CI)	p value
LUTS	3004	3706	0.917 (0.874, 0.963)	< 0.001***
Depression	106	196	0.616 (0.486, 0.780)	< 0.001***
Anxiety	144	297	0.551 (0.451, 0.672)	< 0.001***
Insomnia	90	159	0.645 (0.498, 0.835)	< 0.001***
Dementia	81	131	0.705 (0.535, 0.930)	0.013*
Delirium	86	141	0.694 (0.530, 0.907)	0.007*

**Image 1.** Kaplan–Meier Curve of Psychiatric Disorder Risk in BPH Patients Undergoing Surgery versus Non-Surgical Controls (BPH = Benign Prostatic Hyperplasia)

**Image 2 & 3.** Subgroup Analysis Forest Plot of Outcomes After Surgery versus Controls (BPH = Benign Prostatic Hyperplasia; LUTS = Lower Urinary Tract Symptoms; HR = Hazard Ratio; CI = Confidence Interval)

**Image 1:**
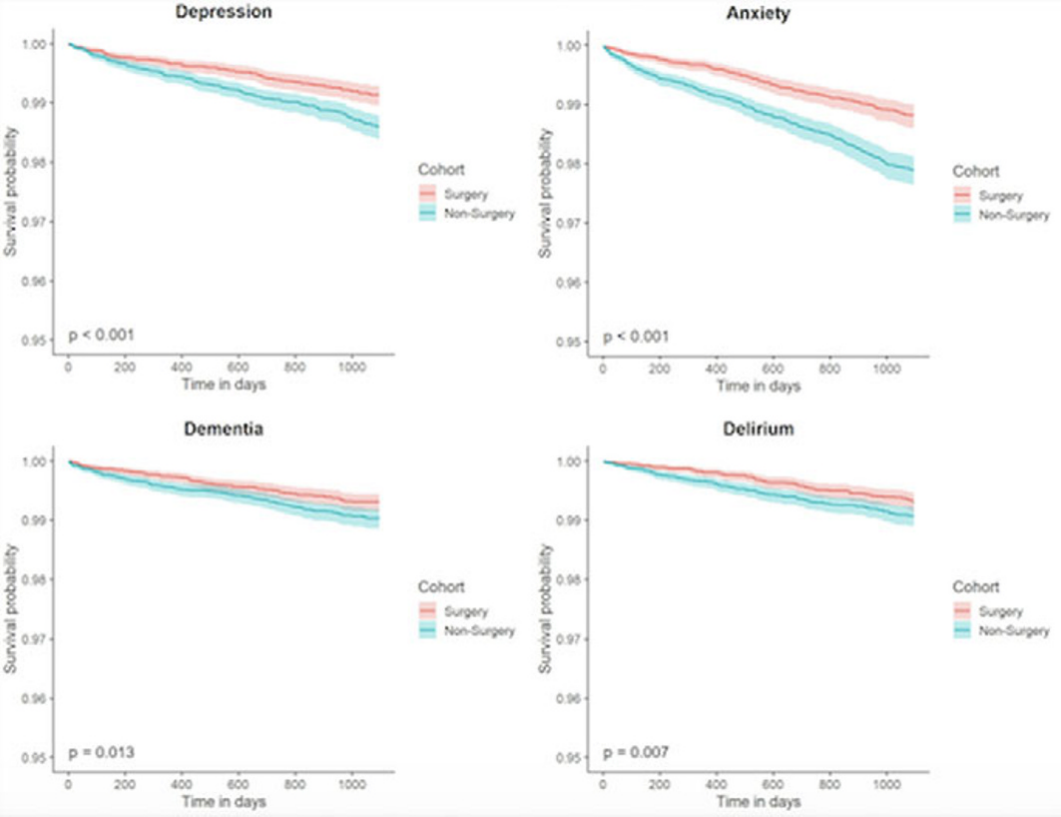
Image 1: Long description.

**Image 2:**
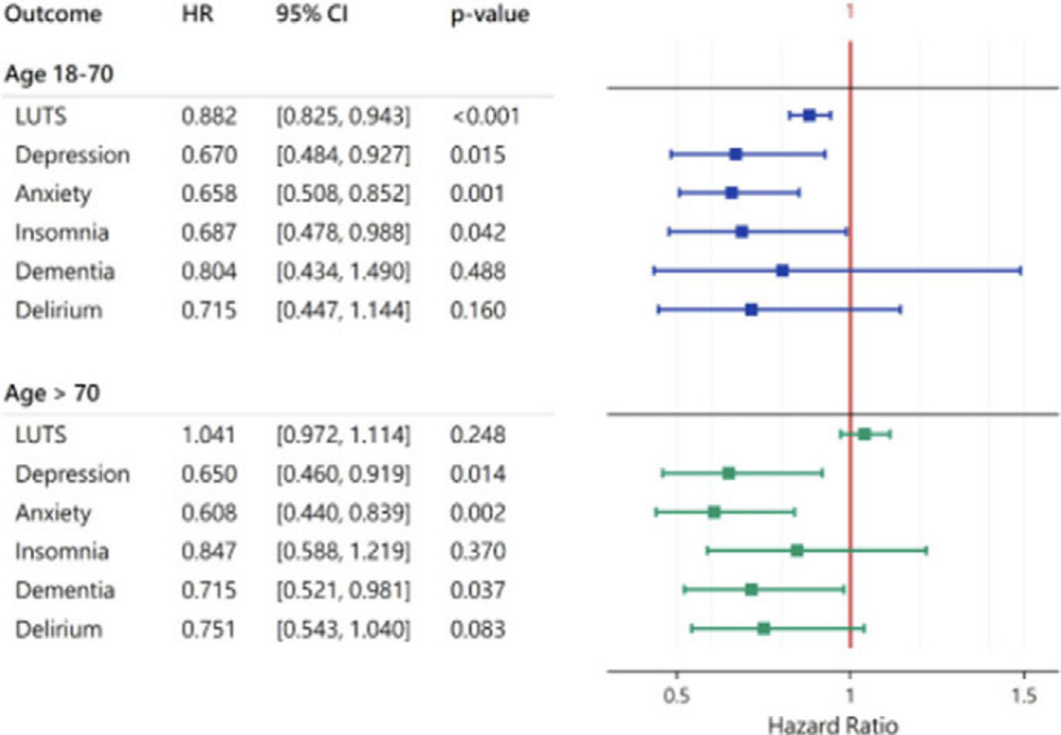
Image 2: Long description.

**Image 3:**
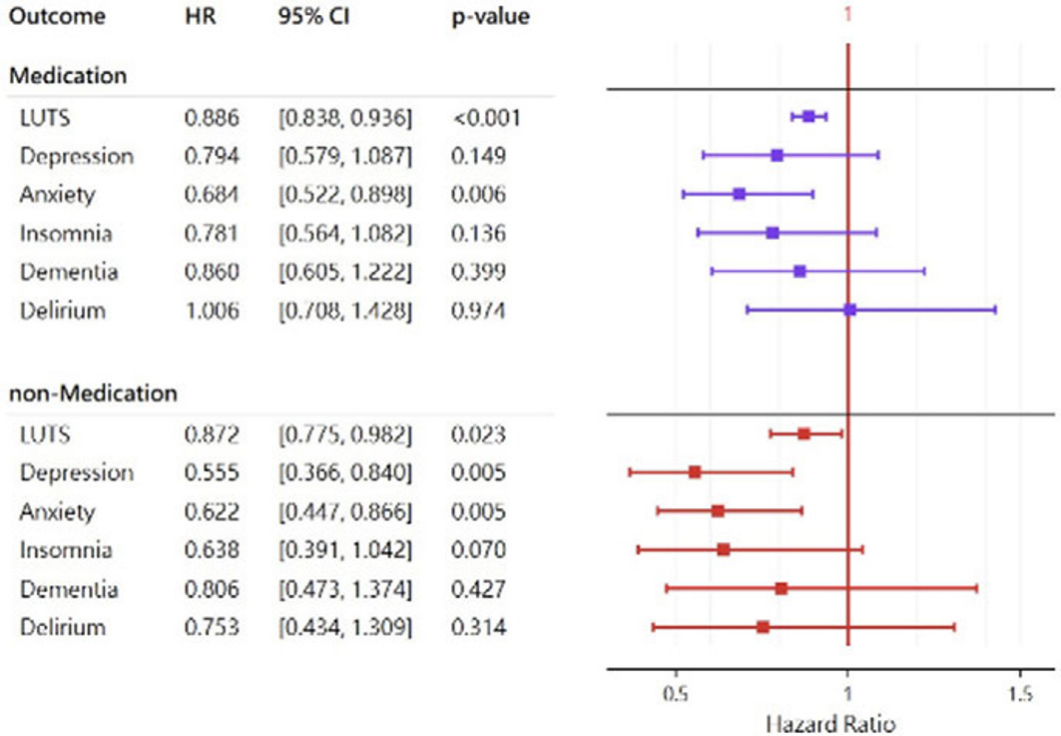
Image 3: Long description.

**Conclusions:**

Surgical treatment for BPH is associated with reduced risks of psychiatric disorders. Beyond relieving urinary obstruction, surgery may confer broader mental health and cognitive benefits.

**Disclosure of Interest:**

None Declared

## Long descriptions

### Table 1. Long description

The table presents data for two groups, Surgery and non-Surgery, each with a sample size N equals 20,323.

* L U T S: Surgery 3004, non-Surgery 3706. Hazard ratio 0.917 (0.874, 0.963). p value less than 0.001.

* Depression: Surgery 106, non-Surgery 196. Hazard ratio 0.616 (0.486, 0.780). p value less than 0.001.

* Anxiety: Surgery 144, non-Surgery 297. Hazard ratio 0.551 (0.451, 0.672). p value less than 0.001.

* Insomnia: Surgery 90, non-Surgery 159. Hazard ratio 0.645 (0.498, 0.835). p value less than 0.001.

* Dementia: Surgery 81, non-Surgery 131. Hazard ratio 0.705 (0.535, 0.930). p value 0.013.

* Delirium: Surgery 86, non-Surgery 141. Hazard ratio 0.694 (0.530, 0.907). p value 0.007.

Navigate back to Table 1..

### Image 1: Long description

A four-panel set of line graphs. Each graph shares the same axes: the y-axis represents Survival probability ranging from 0.95 to 1.00, and the x-axis represents Time in days ranging from 0 to 1000. A legend to the right of each plot identifies two cohorts: Surgery (red line) and Non-Surgery (teal line), both with shaded confidence intervals.

* Top-Left Panel (Depression): The Surgery cohort shows a higher survival probability than the Non-Surgery cohort. Both lines decline gradually, with the Non-Surgery cohort reaching approximately 0.985 and the Surgery cohort reaching 0.992 at 1000 days. The p-value is less than 0.001.

* Top-Right Panel (Anxiety): This graph shows the widest divergence. The Non-Surgery cohort declines more steeply to approximately 0.98, while the Surgery cohort remains higher at approximately 0.99. The p-value is less than 0.001.

* Bottom-Left Panel (Dementia): Both cohorts show very high survival. The Surgery cohort stays above 0.993, while the Non-Surgery cohort ends slightly lower at approximately 0.99. The p-value is 0.013.

* Bottom-Right Panel (Delirium): Similar to Dementia, both cohorts maintain high survival. The Surgery cohort ends at approximately 0.994 and the Non-Surgery cohort at approximately 0.991. The p-value is 0.007.

Navigate back to Image 1:.

### Image 2: Long description

The forest plot is organized into two main horizontal blocks. The x-axis at the bottom represents the Hazard Ratio, ranging from 0.5 to 1.5, with a vertical red reference line at 1.0.

Top Block: Age 18-70 (Blue markers)

* L U T S: H R 0.882, 95% C I [0.825, 0.943], p < 0.001. The marker and error bars are entirely to the left of the red line.

* Depression: H R 0.670, 95% C I [0.484, 0.927], p 0.015. Marker is left of the red line.

* Anxiety: H R 0.658, 95% C I [0.508, 0.852], p 0.001. Marker is left of the red line.

* Insomnia: H R 0.687, 95% C I [0.478, 0.988], p 0.042. Marker is left of the red line.

* Dementia: H R 0.804, 95% C I [0.434, 1.490], p 0.488. Error bars cross the red line.

* Delirium: H R 0.715, 95% C I [0.447, 1.144], p 0.160. Error bars cross the red line.

Bottom Block: Age > 70 (Green markers)

* L U T S: H R 1.041, 95% C I [0.972, 1.114], p 0.248. Marker is slightly right of the red line; error bars cross it.

* Depression: H R 0.650, 95% C I [0.460, 0.919], p 0.014. Marker is left of the red line.

* Anxiety: H R 0.608, 95% C I [0.440, 0.839], p 0.002. Marker is left of the red line.

* Insomnia: H R 0.847, 95% C I [0.588, 1.219], p 0.370. Error bars cross the red line.

* Dementia: H R 0.715, 95% C I [0.521, 0.981], p 0.037. Marker and error bars are left of the red line.

* Delirium: H R 0.751, 95% C I [0.543, 1.040], p 0.083. Error bars cross the red line.

Navigate back to Image 2:.

### Image 3: Long description

The forest plot is divided into two vertical sections: Medication (top, purple markers) and non-Medication (bottom, red markers). The x-axis at the bottom represents the Hazard Ratio (H R) on a scale from 0.5 to 1.5, with a vertical line of null effect at 1.0.

Medication Group:

- L U T S: H R 0.886, 95% C I [0.838, 0.936], p < 0.001. The point estimate and error bars are entirely to the left of the null line.

- Depression: H R 0.794, 95% C I [0.579, 1.087], p = 0.149.

- Anxiety: H R 0.684, 95% C I [0.522, 0.898], p = 0.006.

- Insomnia: H R 0.781, 95% C I [0.564, 1.082], p = 0.136.

- Dementia: H R 0.860, 95% C I [0.605, 1.222], p = 0.399.

- Delirium: H R 1.006, 95% C I [0.708, 1.428], p = 0.974. The point estimate sits directly on the null line.

non-Medication Group:

- L U T S: H R 0.872, 95% C I [0.775, 0.982], p = 0.023.

- Depression: H R 0.555, 95% C I [0.366, 0.840], p = 0.005.

- Anxiety: H R 0.622, 95% C I [0.447, 0.866], p = 0.005.

- Insomnia: H R 0.638, 95% C I [0.391, 1.042], p = 0.070.

- Dementia: H R 0.806, 95% C I [0.473, 1.374], p = 0.427.

- Delirium: H R 0.753, 95% C I [0.434, 1.309], p = 0.314.

Navigate back to Image 3:.

